# Retained surgical sponges: a descriptive study of 319 occurrences and contributing factors from 2012 to 2017

**DOI:** 10.1186/s13037-018-0166-0

**Published:** 2018-06-29

**Authors:** Victoria M. Steelman, Clarissa Shaw, Laurel Shine, Abbey J. Hardy-Fairbanks

**Affiliations:** 1 The University of Iowa College of Nursing, 50 Newton Road, Iowa City, IA 52242-1121 USA; 2 The Joint Commission, 1 Renaissance Boulevard, Oak Brook Terrace, IL 60181 USA; 3 The University of Iowa Carver College of Medicine, 451 Newton Road, Iowa City, IA 52242 USA

**Keywords:** Sponges, Gossypiboma, Adverse event, Patient safety, Surgery, Labor and delivery, Obstetrics

## Abstract

**Background:**

Unintended retention of foreign bodies remain the most frequently reported sentinel events. Surgical sponges account for the majority of these retained items. The purpose of this study was to describe reports of unintentionally retained surgical sponges (RSS): the types of sponges, anatomic locations, accuracy of sponge counts, contributing factors, and harm, in order to make recommendations to improve perioperative safety.

**Methods:**

A retrospective review was undertaken of unintentionally RSS voluntarily reported to The Joint Commission Sentinel Event Database by healthcare facilities over a 5-year period (October 1, 2012- September 30, 2017). Event reports involving surgical sponges were reviewed for patients undergoing surgery, invasive procedures, or child birth.

**Results:**

A total of 319 events involving RSS were reported. Sponges were most frequently retained in the abdomen or pelvis (50.2%) and the vagina (23.9%). Events occurred in the Operating Room (64.1%), Labor and Delivery (32.7%) and other procedural areas (3.3%). Of the events reported, 318 involved 1 to 12 contributing factors totaling 1430 in 13 different categories, most frequently in *human factors* and *leadership*. In 69.6% of reports, the harm was an *unexpected additional care or extended stay*. *Severe temporary harm* was associated with 14.7% of the events. One patient died as a result of the retained sponge.

**Conclusions:**

Because of the complexity of perioperative patient care, the multitude of contributing factors that are difficult to control, and the potential benefit of radiofrequency sponge detection, we recommend that this technology be considered in areas where surgery is performed and in Labor and Delivery.

## Background

Unintended retentions of a foreign object after surgery (e.g. sponge, needle, and instrument) (URFO) remain the sentinel events most frequently reported to The Joint Commission (TJC) [1] (See list of abbreviations). Although these events have happened in other invasive procedures, URFOs are estimated to occur in 1:5500 surgeries [2]. These serious adverse events have resulted in patient harm involving reoperation [3, 4], readmission/prolonged hospital stay [3, 4], infection or sepsis [3], fistulas/ bowel obstructions [3], visceral perforation [3], and death [3]. Cotton gauze sponges account for 48–69% of retained surgical items [2–4], and result in more serious tissue reaction than metal fragments.

The Joint Commission requires that accredited facilities conduct a root cause analysis (a process for identifying the factors that underlie variation in performance) when a sentinel event, such as a retained surgical sponge (RSS), occurs. The goals of this examination are four-fold: 1) to provide a positive impact on improving patient care and preventing sentinel events, 2) focus the attention of the hospital on factors that contributed to the event, 3) increase general knowledge about these events and strategies for prevention, and 4) maintain public confidence in accredited hospitals [5]. However, the root cause analysis focuses on a single, relatively rare event, and thus provides a very, limited view on how all of these events can be prevented. Examining a large dataset of sentinel events provides broader and more in-depth knowledge of the context in which RSS occur. This knowledge is needed to design safer processes of care and improve patient safety.

## Methods

The purpose of this study was to describe reports of unintentionally RSS, including: the types of sponges, anatomic locations, accuracy of sponge counts, contributing factors, and harm. For this descriptive study, we retrospectively reviewed de-identified events reported to The Joint Commission Office of Quality and Patient Safety from October 1, 2012 through September 30, 2017. Events were either voluntarily reported by TJC-accredited organizations or reported by other entities and determined to meet the definition of Sentinel Event.

Inclusion criteria were: a) a surgical sponge, b) an event meeting TJC definition of sentinel event, and c) an event meeting TJC definition of unintended retention of a foreign object (URFO). A surgical sponge was defined as cotton material (e.g. laparotomy sponge, raytec, cottonoid, towel, and kerlix) inserted during an invasive procedure to absorb fluids or isolate tissue, with the intention of removing the absorbent material prior to completion of the procedure. The Joint Commission definition of sentinel event includes “unintended retention of a foreign object in a patient after an invasive procedure, including surgery.” [5] The Joint Commission definition of a URFO is an object that is retained after skin closure has occurred following an invasive procedure. [5] Exclusion criteria were: intentionally placed packing intended to be removed at a later date/time (e.g. vaginal packing, sponges intentionally packed for damage control laparotomies).

Reporters described events that reached the patient and did or did not cause harm. Managers (or their designees) of the reporting units (e.g. operating room, labor and delivery, cardiac catheterization) and safety department reviewed the accuracy of the harm scores and identified contributing factors (e.g. communication, staff inattention) from categories in TJC standardized list. Reports were reviewed and edited as necessary by staff members of TJC’s Office of Quality and Patient Safety. Subsequently, reports were placed in the sentinel event database. A search was performed of reports in the database in the event category “unintended retention of a foreign body”, and event subcategory of “sponge”. These data were reviewed by a TJC staff member independent of the researchers, who de-identified the data, and redacted any information which could compromise confidentiality of patients or facilities. Events in other subcategories of URFO were manually reviewed to determine if a RSS was described. Event reports were then reviewed by three researchers (VS, CS, LS) to identify type of sponge, anatomical location, surgical specialty, department, contributing factors, outcomes of surgical counts, use of adjunct technology, and patient harm. When questions occurred, categorization was determined by consensus. Data were described as frequencies and percents.

## Results

The data included 319 reports of RSS. The type of sponge was identified in 159 (49.8%) reports (See Table 1). Most of these (52.2%; *n* = 83 of 159) were laparotomy sponges. These radiopaque sponges are usually 18 in. square and are routinely used in thoracic and abdominal surgery (including Cesarean sections). The second most frequently identified RSS was a 4 in. by 4 in. or 4 in. by 8 in. sponge (34.0%; *n* = 54 of 159). Of these, either the reporters indicated or we were able to deduce from the narrative reports that 81% (*n* = 44 of 54) were radiopaque. Eleven reports (6.9% of 159) were of the retained towels. Cottonoids, small radiopaque neurosurgical patties, were identified in 3.1% (n = 5 of 159) of reports.

**Table 1:** Type of Unintentionally Retained Sponges (*N* = 319)

Type of sponge	Sponges		Radiopaque	
	N	%	N	%^a^
Laparotomy	83	26.0	83	100.0
4 X 4/ 4 X 8/ raytec	54	16.9	44	81.5
Towel	11	3.4	0	0.0
Cottonoid	5	1.6	5	100.0
Kerlix	2	0.6	0	0.0
Peanut	1	0.3	1	100.0
Tonsil	1	0.3	1	100.0
4 × 10	1	0.3	1	100.0
2 × 4	1	0.3	0	0.0
Unknown	160	50.2	64	40.0
Total	319	100	199	62.4

^a^Percent of radiopaque by number of sponges

### Anatomic location of sponges

Of the 319 reports, 305 identified the anatomical location of the sponge, ranging from the head to the leg (See Table 2). The majority (50.2%, *n* = 153 of the 305) were in the abdomen or pelvis. One of these was retained in the uterus. The vagina was the second most frequently reported site (23.9%, *n* = 73 of 305).

**Table 2:** Location of Unintentionally Retained Sponges (*N* = 305)

Location	N	%
Abdomen/pelvis	153	50.2
Vagina	73	23.9
Chest/mediastinum	26	8.5
Breast/Pacemaker/ICD pocket	14	4.6
Back	11	3.6
Mouth/Airway	7	2.3
Shoulder	5	1.6
Axilla	3	1.0
Leg/gluteal region	3	1.0
Intracranial	2	0.7
Eye	2	0.7
Neck	2	0.7
Nasal Cavity	1	0.3
Arm	1	0.3
Scrotum	1	0.3
Hip	1	0.3
Total	305	100.0%

The chest or mediastinum was identified in 8.5% of these reports (*n* = 26 of 305). In 4.6% of RSS (*n* = 14 of 305), sponges were retained in the breast or the pocket made to insert an internal pacemaker/defibrillator. Three of the reports (1.9% of 305) involved sponges placed in the throat during otolaryngology or dentistry procedures. The intent was that these sponges be removed prior to endotracheal extubation.

### Department involved

**Fig. 1:**
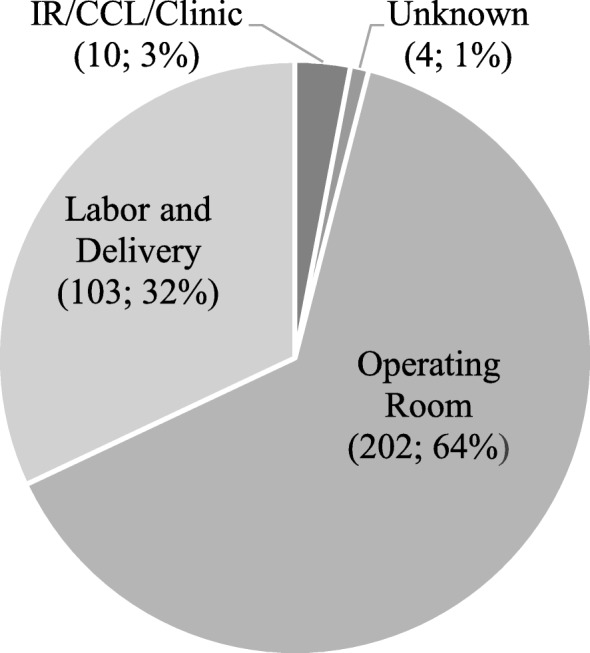
Department where sponge was unintentionally retained (*N* = 319) (n; %). *Note*. IR = Interventional Radiology; CCL = Cardiac Catheterization Lab

The department involved in the retained sponge was identified or could be deduced in 315 reports (See Fig. 1). Most (64.1%; *n* = 202 of 315) were retained after surgeries in the Operating Room; one third (32.7%; *n* = 103 of 315) were retained in Labor and Delivery. Ten of the 315 reports (3.3% of 315) involved sponges retained during surgical procedures in other invasive procedure areas (Interventional Radiology or Cardiac Catheterization Lab (*n* = 9), Urology Clinic (*n* = 1)).

The type of surgical procedure was reported in 92.2% (*n* = 294 of 319) of the cases. Nearly half of retained sponges occurred in obstetric and gynecologic procedures: (obstetrics - 34.7% (*n* = 102 of 294); gynecologic - 10.2% (*n* = 30 of 294); urogynecologic - 1.7% (*n* = 5 of 294)). General surgery was involved in 25.5% (*n* = 75 of 294) and cardiothoracic procedures were involved in 11.9% (*n* = 35 of 294) of cases. Trauma procedures were involved in 1.7% (*n* = 5 of 294) of the cases.

### Sponge counts

Reporters indicated that a sponge count was performed in 77.4% (*n* = 247 of 319) of reports. When the count was performed, it was reported as being correct 80.6% (*n* = 199 of 247) of the time. Counts were not performed in 8.8% (*n* = 28 of 319) of the reports, of which 50% (*n* = 14 of 28) were identified as emergent.

Of the reports of retained sponges in general surgery procedures, sponge counts were performed in 90.0% (*n* = 72 of 80), and 86.1% (*n* = 62 of 72) of those counts were considered correct. Of the sponges retained in the abdomen/pelvis during obstetric and gynecologic procedures, counts were known to be performed in 72.3% (*n* = 47 of 65) of the cases with 68.1% (*n* = 32 of 47) considered correct. Counts were performed in 58.3% (*n* = 35 of 60) of vaginal deliveries of which 94.3% (*n* = 33 of 35) were considered correct.

### Sponge detection technology

Radiofrequency (RF) or radiofrequency identification (RFID) sponge detection technology was used in nine reports of retained sponges and detected sponges in eight cases. In six of these, the technology was used after the incision was closed and the sponge was removed during the same procedure. In one event, the sponge count was correct, the technology identified a retained sponge and alarmed, but the detection was ignored. In another, the scan was performed in the Postanesthesia Care Unit. In the only report where the technology was used and did not detect the sponge, a kerlix was retained during a trauma surgery and found during a planned secondary operation. Kerlix does not contain a RF chip or a radiopaque marker and the hospital policy was not followed. Of the eight cases in which the RF or RFID sponge detection identified a sponge, the sponge count was correct in six (75% of 8). In five other cases, sponge detection technology was noted to be available in the setting, but not used.

### Contributing factors

A total of 1430 contributing factors were assigned by TJC staff based on root cause analyses. These were clustered into 13 categories with 63 subcategories (See Table 3). Of the events, 318 involved 1 to 12 contributing factors, each event could be assigned multiple subcategories under a single main category. One report lacked the detail to identify a root cause. The Joint Commission category, *Human Factors* (interactions between humans), was the most frequently identified cause of a RSS (29.2%, *n* = 417 of 1430) with subcategories *medical staff peer review* and *medical staff credentialing* the most frequent subcategories (*n* = 126 of 417). The category of *other human factors issues* was reported in 120 events (8.4%; 120 of 1430).

**Table 3:** Contributing Factors to Retained Surgical Sponges

Human Factors (n = 417)	
Medical staff peer review/credentialing	126
Staff orientation/in-service education	94
Competency assessment	49
Staff supervision	13
Resident supervision	9
Staffing levels/skill mix	6
Other human factor issues	120
Leadership (n = 394)	
Compliance with policies & procedures	205
Policies & procedures	129
Organizational culture	31
Directing departments/services	11
Nursing leadership	3
Medical staff - Other	3
Other leadership issues	12
Communication (*n* = 330)	
With physician	153
Among staff	92
Oral communication	54
Written/electronic communication	14
With administration	13
Other communication issues	4
Operative Care (*n* = 108)	
Other operative care issues	69
Operative care planning	37
Other	2
Assessment (*n* = 82)	
Adequacy of assessment	56
Patient observation	19
Scope or timing of reassessment	4
Care decisions	3
Physical Environment (n = 33)	
Equipment management	22
Emergency management	2
Other environmental issues	9
Information Management (*n* = 25)	
Technical systems	10
Availability of information	8
Medical records	6
Patient identification	1
Performance Improvement (*n* = 21)	
Data collection	12
Other PI issues	9
Other	20
Grand Total^a^	**1430**

^a^No root cause was identified in 1 event report

Issues in *Leadership* (27.6%, *n* = 394 of 1430) and *Communication* (23.1%; 330 of 1430) were the next most frequently identified categories of contributing factors. The subcategories *compliance with policies and procedures* (14.3%, *n* = 205 of 1430), and *policies and procedures (9.0%; n = 129 of 1430)* were frequently identified*. Communication with physician* (10.7%, *n* = 153 of 1430), and *communication among staff* (6.4%, *n* = 92 of 1430) were frequently identified. Of the remaining root causes identified, *adequacy of patient assessment* (3.9%; *n* = 56 of 1430) was the most frequent.

### Discovery

The length of time for discovery of the retained sponges was known in 76.2% (*n* = 243 of 319) of the cases (See Table 4). Less than one-fifth (16.5%, *n* = 40 of 243) were identified while the patient was still in the operating/procedure room following incision closure or procedure completion. Over one-third (34.2%; *n* = 83 of 243) of the sponges were identified while the patient was hospitalized following the surgical procedure, regardless of length of stay. The remaining 49.4% (*n* = 120 of 243) of the sponges were identified after the patient was discharged from the facility, with 15.2% (*n* = 37 of 243) identified within the first 7 days, 16.0% (*n* = 39 of 243) within 7–30 days, and 18.1% (*n* = 44 of 243) more than 30 days of hospital discharge. Three cases (1.2%) noted that it took over one year to identify the RSS.

**Table 4:** Timeframe for Discovery (N = 243)

Timeframe	N	%
Operating Room, post-closure	40	16.5
Hospitalization, post-OR discharge	83	34.2
Within 7 days of hospital discharge	37	15.2
Greater than 7 days post-discharge	39	16.0
Greater than 30 days post-discharge	44	18.1
Unknown	76	16.5
Total	243	

### Harm

All 319 reports included a harm score assigned by TJC staff members (See Table 5). The majority (69.6%; 222 of 319) were categorized as *unexpected additional care/extended stay*, followed by *severe temporary harm* (14.7%; 47 of 319). There was one *death* related to a retained sponge in the airway during a non-emergent laryngoscopy, one instance of *permanent harm* related a retained laparotomy sponge during an urgent exploratory laparotomy, and two instances of *permanent loss of function* related to an unknown retained sponge during a bowel resection and a retained laparotomy sponge during an emergent Cesarean-section.

**Table 5:** Harm Attributed to the Retained Sponge (*N* = 319)

	N	%
Unexpected Additional Care/Extended Stay	222	69.6
Severe Temporary Harm	47	14.7
Permanent Loss of Function	2	0.6
Psychological Impact	2	0.6
Permanent Harm	1	0.3
Death	1	0.3
Other	44	13.8
Total	319	100.0%

## Discussion

This study identifies that unintentionally RSS continue to be a significant problem and provides evidence about the context in which sponges were retained in the Operating Room, Labor and Delivery, and other areas where surgical procedures are performed. A total of 1430 contributing factors were identified with 79.8% (*n* = 1141 of 1430) relating to human factors, leadership, and communication alone. These findings provide additional knowledge and support the complexity of patient care issues identified in a Healthcare Failure Mode and Effects Analysis (HFMEA) examining prevention of RSS [6]. This proactive risk assessment identified 57 different failure cause combinations, with 43 rising to the level of criticality requiring control. In this HFMEA, the most common causes of potentially retained sponges were distraction (21%), multi-tasking (18%), and time pressure/emergency (18%). These potential causes are very difficult if not impossible to control [6].

Sponge counts were reported as having been completed in 77.4% of cases of retained sponges. When a count was performed, it was reported as correct in 80.6% of the events involving retained sponges. This was higher in general surgery (86.1%). These findings are consistent with previous research that found the sensitivity of the surgical count to be 77.2% and a review of closed claims in which 88% of retained surgical items occurred when the count was correct [3, 7]. Fifty percent of the events in which a sponge count was not performed (*n* = 14 of 28) were emergent procedures. In these cases, the sponge count would not be considered the highest priority for patient care.

The variety of contributing factors and the failure of the sponge count to provide effective prevention warrants consideration of technological solutions. The traditional technology used for this purpose has been intraoperative radiographs. This has been routinely done when a sponge count is not performed or incorrect [8]. However, using intraoperative radiographs for detecting retained surgical items has been found to be only 67% sensitive [2]. The Association of periOperative Registered Nurses acknowledges that the collective evidence suggests that the sensitivity and specificity of manual counting and radiograph screening is insufficient to prevent RSI (retained surgical items) (p. 406) [8]. Professional associations recommend considering other adjunct technology [8–10].

### Radiofrequency sponge detection

Two adjunct technologies (low frequency radiofrequency (RF) and radiofrequency identification (RFID) detect the presence of a retained surgical sponge. Low frequency radiofrequency (RF) sponge detection has been found to be 100% sensitive in identifying retained sponges [11]. The RF detection technology includes chip in the sponge that is detected by a handheld wand or wands built into an underbody mat. It can be safely used to scan in patients with pacemakers and internal defibrillators if the cardiac device is set to asynchronous mode [12]. An observational study comparing before and after implementation of the RF technology found that using the technology resulted in a 79.6% reduction in time spent searching for sponges and 71.3% reduction in unreconciled counts, resulting in a significant reduction of estimated costs [13]. A clinical trial found that RF technology reconciled 35 miscounts and resulted in no retained sponges during an 18-month period of time [14]. A study of 2148 emergency surgeries found that counting was not performed in 45.5% of cases. The technology prevented 11 retained sponges. No RSS occurred. The authors concluded that when using the RF sponge detection, it should be used regardless of the outcome of the sponge count and that there is no need for radiographs, even when the count is not performed or is incorrect [15].

The University HealthSystem Consortium (now Vizient) conducted a cost-benefit analysis of the RF sponge detection system and found that the savings in X-rays and time spent in the operating room, and the avoidance of medical and legal costs outweighed the expenses involved in using the RF technology [16].

In the reports we reviewed, RF technology used correctly may have prevented up to 97.2% (*n* = 310 of 319) of retained sponges, including 11 towels. It would not have prevented 9 retained sponges, which were of materials not detectable by this technology. The technology would not prevent retained cottonoids (*n* = 5) or peanuts (*n* = 1). The cases of retained kerlix gauze (*n* = 2), and a non-radiopaque sponge obtained from an anesthesia kit (*n* = 1) would not have a RF chip and would not have been prevented. In one of these cases, the kerlix was cut and a fragment retained. It is worth noting that kerlix is a dressing, not a surgical sponge, has no radiopaque marker, and its use as a sponge is not recommended [8, 9]. Furthermore, guidelines recommend that only radiopaque sponges be used and that sponges never be cut [8, 9].

### Vaginal sponges

In this current study, the vagina was the second most frequent site of a retained sponge, comprising 23.9% of retained sponges. Of these, 82.2% (*n* = 60 of 73) occurred following vaginal delivery. These findings are similar to those identified in previous research showing that 22–32% of retained sponges are left in the vagina [3, 17]. Lutgendorf et al. (2011) found that a sponge was retained during 1 in 5000 vaginal deliveries [18]. In our current study, the documentation of vaginal sponge discovery was identified in 82% (*n* = 49 of 60) of the cases, with only 18% (*n* = 9 of 49) of sponges being identified while the patient was still hospitalized. The majority of the vaginal sponges (39%, *n* = 19 of 49) were not identified until 7 to 30 days post-delivery, with 6% (*n* = 3 of 49) not identified until greater than 1 month post-delivery. Although many of these sponges may never cause permanent harm, there is still substantial patient risk. Retained vaginal sponges can lead to increased healthcare treatment related to pain and infection [19] and have even led to maternal deaths related to systemic infection [20]. Multiple hospitals have successfully implemented procedures to reduce vaginal sponge retention including use of formal counts, improving teamwork and communication, and use of large radiopaque sponges to be identified with x-ray or radiofrequency technology [18, 19, 21, 22].

Vaginal delivery is often treated as less of a surgical procedure and thus does not have the same degree of patient safety procedures such as sponge or instrument counts, “time outs” or check lists that have shown such success in the operating theatre. Nonetheless, vaginal delivery is a surgical procedure that is fraught with opportunity for preventable error. Data in this study, as in others, demonstrate that the national initiation of sponge counts and radiofrequency technology on labor and delivery units would decrease maternal morbidity from preventable retained sponges.

### Recommendations

Based upon the results of this study in the context of previous research, several recommendations can be made to minimize the risk of RSS. First, a methodologic wound exploration should be performed to retrieve all sponges prior to wound closure or completion of the procedure. For procedures involving the vagina, a vaginal sweep should be performed. Kerlix should not be used as a sponge. And, sponges should not be cut. Sponges identified and removed after the incision is closed/procedure completed, prior to transfer from the operating/procedure room, should be considered either a sentinel event, or a near miss. These events should be reported internally and investigated.

Because of the multitude of contributing factors that have led to a RSS, and the published accuracy and potential benefit of the RF sponge detection, we recommend that this technology be seriously considered in areas where surgery is performed and in Labor and Delivery. Laparotomy, episiotomy, 4 X 4 s and towels should all include a RF chip. Small sponges unavailable with a RF chip (cottonoids, peanuts, and kittners) should be counted. If the patient has a cardiac pacemaker or defibrillator, it should be set on asynchronous mode. Policies should be clear. Education on the use of the technology should be provided for staff and physicians in operative and other invasive procedures and Labor and Delivery. Routine monitoring of compliance with manufacturers’ written instructions for use should be included in the facility’s quality performance measures, and regular feedback provided to personnel.

### Limitations

This study has limitations. We used a retrospective observational study design. Reporting sentinel events to TJC, in most cases, is voluntary and may have resulted in selection bias. Incidence and prevalence of RSS cannot be determined because of the voluntary nature of reporting and the lack of a denominator. The Joint Commission definition of a URFO is “an object that is retained after skin closure has occurred following an invasive procedure” [23]. Some hospitals use the National Quality Forum definition of serious reportable event which states, “and the patient has been taken from the operating/procedure room” (p. B-4) [24]. Thus, the events discovered in the operating/procedure room are under-reported. Hospitals may be unaware of sponges removed from the vagina outside of the hospital setting, so these events were likely under-reported. Events were identified using keyword searches using specific terms. Although the search function includes approximate text matches in the search results, the list of keywords used in the search is not comprehensive, so some sentinel events might not have been identified.

Some reports were incomplete or information was redacted for confidentiality, and the missing information would have been valuable. Lastly, the categories of harm used by TJC changed during this study period. Reporters rated harm lower than the definitions would indicate, at times considering reoperation no harm. When possible, patient safety reviewers from TJC reclassified the harm ratings.

## Conclusions

This study provides new insight into the ongoing problem of RSS, describing 319 reported events over a five-year period. This is the largest sample of RSS we have seen in published literature. The knowledge gained is much more comprehensive than could be attained by conducting a single root cause analysis in a healthcare facility. The results provide evidence about the context in which sponges were retained in the Operating Room, Labor and Delivery, and other areas where surgical procedures are performed. The vast number of contributing factors identified make refinement of current processes very difficult to do achieve and likely ineffective to prevent all RSS. We recommend the addition of sponge detection technology to verify that no sponge remains in the patient prior to discharge from the operating/procedure room.

## Abbreviations

RF: Radiofrequency
RFID: Radiofrequency identification
RSS: Retained surgical sponge(s)
TJC: The Joint Commission
URFO: Unintended retention of a foreign object
